# Nod-Like Receptors: Cytosolic Watchdogs for Immunity against Pathogens

**DOI:** 10.1371/journal.ppat.0030152

**Published:** 2007-12-28

**Authors:** Jean-Claude Sirard, Cécile Vignal, Rodrigue Dessein, Mathias Chamaillard

**Affiliations:** University of British Columbia, Canada

## Abstract

In mammals, tissue-specific sets of pattern-recognition molecules, including Nod-like receptors (NLR), enable concomitant and sequential detection of microbial-associated molecular patterns from both the extracellular and intracellular microenvironment. Repressing and de-repressing the cytosolic surveillance machinery contributes to vital immune homeostasis and protective responses within specific tissues. Conversely, defective biology of NLR drives the development of recurrent infectious, autoimmune and/or inflammatory diseases by failing to mount barrier functions against pathogens, to tolerate commensals, and/or to instruct the adaptive immune response against microbes. Better decoding microbial strategies that are evolved to circumvent NLR sensing will provide clues for the development of rational therapies aimed at curing and/or preventing common and emerging immunopathologies.

## Introduction

**Figure 1:**
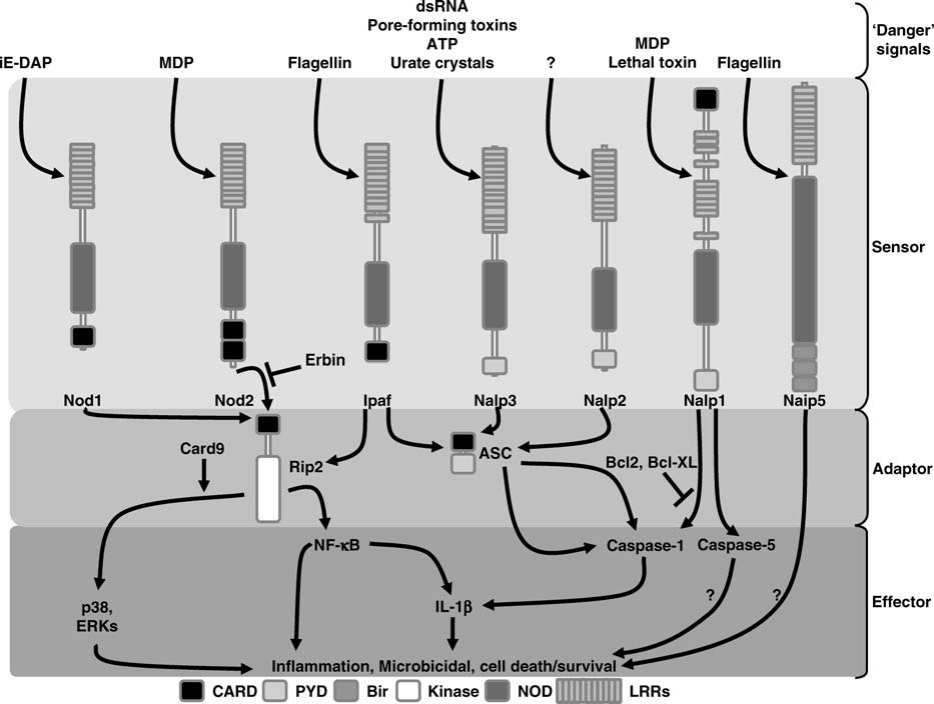
Intracellular Debugging of the NLR Signaling Pathways A schematic overview of the major NLR signaling pathways in innate immunity is depicted. Upon detection of their agonists, NLR likely oligomerize through the NOD domain and recruit at least three specific adaptors, including RIP2, CARD9, and ASC. Several modulators of NLR signaling have been recently identified, such as Erbin, Bcl2, and Bcl-xl. The maturation of IL-1β by the inflammasome illustrates the interplay between NLR (i.e., NALP1–3, NAIP5, and IPAF) and other PRM, such as TLR. Better understanding of the spatio-temporal engagement and/or repression of specific NLR might open new avenues for therapeutical intervention.

Mammals face life-threatening signals and have the double-edged challenge of eliminating infectious agents and tolerating their flora, especially in the gastrointestinal tract. In mammals, the combination of germ-line encoded pattern-recognition molecules (PRM), including Toll-like receptors (TLR) and Nod-like receptors (NLR), plays an essential role in detecting a diversified set of extracellular and intracellular “danger” signals that primarily originate from microbes (so-called microbial-associated molecular patterns [MAMP]) [1,2]. MAMP are highly conserved microbial-derived molecules shared by both pathogens (in which they are designated as PAMP [pathogen-associated molecular patterns]) and commensals, such as lipopolysaccharides, carbohydrates (including peptidoglycans [PGN]), flagellins, nucleic acids, and peptidic and lipidic structures [3]. Originally identified in the fruit fly and plants, the membrane-bound receptors TLR sense MAMP through their extracellular domain, whereas NLR detect signals inside the cells. Upon recognition of their specific MAMP (Table 1), NLR drive innate and adaptive responses and participate in homeostasis within various host tissues through the activation of transcription factors and downstream effectors, such as mitogen-activated protein kinase (MAPK) (Figure 1) [4–9]. Recent studies emphasized major contributions of NLR in microbial pathogenesis and mammalian immunity. Herein, we summarize the mechanisms of microbial detection by NLR, the NLR-mediated immune signaling, the crosstalk between NLR–NLR and NLR–TLR in mammals, and the strategies used by pathogens to circumvent NLR signaling. Lastly, we will discuss the pathophysiological implications of both NLR and TLR in human diseases, because mutations in NLR- and TLR-encoding genes have been linked to chronic inflammatory diseases, resistance and susceptibility toward a panel of infectious agents, and/or autoimmunity.

**Table 1:**
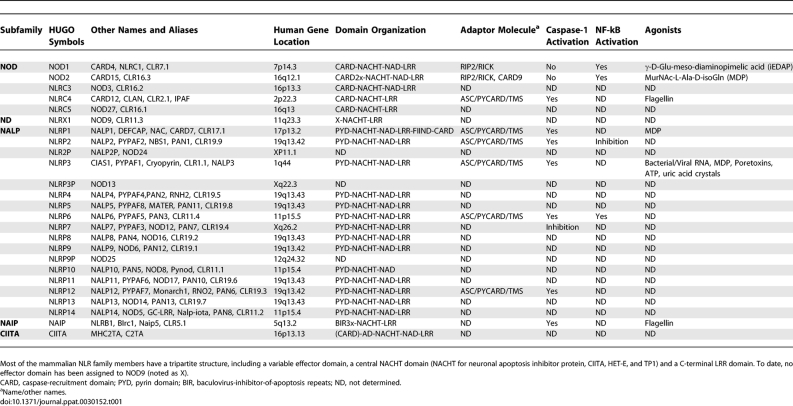
NLR, MAMP, and “Danger” Signals

## NLR Are Cytosolic Biosensors for Both Intra- and Extracellular Microbes

Similarly to the superfamily of plant disease-resistance proteins [10], the structure of NLR, also referred as caterpillers, is composed of a N-terminal effector domain, a central oligomerization domain (called NACHT for neuronal apoptosis inhibitor protein, CIITA, HET-E, and TP1), and a C-terminal collection of leucine-rich repeats (LRR) [4–7]. A set of about 23 mammalian NLR has been recently identified by in silico mining of genomic databases for proteins with homology to the apoptosis regulator Apaf-1. NLR are classified accordingly to their effector domains, caspase-recruitment domain (CARD) for nucleotide-binding oligomerization domain protein (NOD), the pyrin domain (PYD) for NALP, and the baculovirus-inhibitor-of-apoptosis repeats (BIR) for NAIP (Figure 1 and Table 1). The effector modules CARD and PYD belong to the death-domain family and define the specificity of the cellular response by activating multiple signaling pathways through homophilic and heterophilic protein-protein association.

### 

#### The NOD-dependent signaling pathway.

Bacterial PGN is a parietal structure found in all proteobacteria, and both NOD1 and NOD2 have been identified as sensors of fragments derived from PGN, namely muramyl dipeptide (MDP) for NOD2 [11–13] and γ-D-Glu-*meso*-DAP (iE-DAP) for NOD1 [14,15]. Upon recognition of MDP or iE-DAP, the CARD-containing serine/threonine kinase RIP2 (also known as RICK, CARDIAK, CCK, and Ripk2) engages inflammatory and antimicrobial responses independently of TLR [16–18]. The NOD1- and NOD2-dependent immune response can be triggered by invasive bacteria that replicate inside cells, such as Listeria monocytogenes [16,17,19] (Figure 1). Whereas RIP2-deficient mice have increased susceptibility to systemic infection by L. monocytogenes [18], NOD2-deficient mice showed susceptibility to oral (but not systemic) listeriosis [13]. Extracellular bacterial pathogens can also be sensed by NOD1 and NOD2 through the intracellular delivery of muropeptides by either their type III or IV secretion apparatus [20,21]. These secretion machines are molecular syringes that form transport channels through the membrane of target cells to deliver virulence factors into cytosol. However, the mechanisms by which muropeptides are injected intracellularly remain elusive. Notably, NOD1 is required for the innate immune response to Helicobacter pylori, a major agent of gastric ulcer [20]. Conversely, bacterial mutants deficient in PGN synthesis or type IV secretion system have impaired ability to trigger NOD1-mediated responses in epithelial cells of the stomach [20,22].

More recently, CARD9 has been identified to physically interact with NOD2 and RIP2 to selectively synergize the MDP-induced activation of MAPK but not of nuclear factor–κ B (NF-κB) (Figure 1), providing a mechanism by which CARD9 might control innate immune response toward intracellular pathogens [23]. CARD9 is a CARD-containing adaptor which modulates the development of T(H)-17 response [24] and lacks the C-terminal, membrane-associated guanylate kinase domain. Similarly to RIP2-deficient mice, CARD9-deficient mice are susceptible to systemic infections by L. monocytogenes [23]. Because CARD9 is involved in Dectin1-mediated response to fungal infection [25], the physiological role of NOD1 and NOD2 in response to fungi is now eagerly awaited. Lastly, given that CARD9 is also required for TLR3- and TLR7-dependent signaling pathways [23], further work is now warranted to assess the physiological role of CARD9 in immunity to viruses.

#### The inflammasome.

The inflammasome is an inflammatory caspase-activating complex, which contain at least caspase-1 and -5, ASC, NALP1-3, IPAF, and NAIP5 [26]. Notably, caspase-5 is recruited by NALP1, but not NALP2 and NALP3, through homophilic CARD–CARD interactions (Figure 1) [26,27]. However, the physiological role of NALP2 and caspase-5 remains poorly understood [28]. Caspase-1, also known as IL-1–converting enzyme (ICE), is a protease involved in pyroptosis, a recently described form of inflammatory programmed cell death, and is essential for the processing of immature pro-inflammatory cytokine IL-1β and the related members IL-1α, IL-18, and IL-33 [29]. The IL-1β is instrumental to initiate and/or amplify innate and adaptive immunity toward pathogens [28]. Caspase-1 activation is also involved in the cleavage of the MyD88-like adaptor Mal [30] and in membrane biogenesis by promoting cell survival following toxin-induced membrane permeabilization [31]. The contribution of caspase-1 and NALP/NAIP in host–pathogen interaction has been analyzed in vivo by using animal models of infection or in vitro by using cytokine and viability assays with monocytes/macrophages. Caspase-1^−/−^ mice, which are inoculated nasally by the agent of bacillary dysentery *Shigella flexneri* cannot trigger IL-1β–dependent acute inflammation, thereby resulting in exacerbated infection [32]. Interestingly, a positive correlation was defined for *S. Typhimurium* and S. flexneri between their capacity to promote macrophage death and their virulence in mice. This effect has been proposed to depend on the inflammasome [26,33].

Sensing of several extracellular and intracellular pathogenic bacteria is associated to the secretion of IL-1β by activating caspase-1 through at least five NLR, namely ICE protease-activating factor (IPAF), NALP-1, −2 and −3, and NAIP5 (Figure 1 and Table 1). Containing a N-terminal PYD and a C-terminal CARD, the death-fold–containing adaptor apoptosis-associated speck-like protein containing a CARD (ASC) acts as a molecular bridge between NALP1-3, IPAF, and caspase-1 [26] (Figure 1). In vivo, NALP1 senses the Bacillus anthracis lethal toxin, which is delivered in the cytoplasm by receptor-mediated endocytosis [34]. The bacterial PGN component MDP activates both the NALP1- and NALP3-dependent inflammasome [35,36]. NALP3 is also able to detect a large variety of additional microbial signals (such as microbial RNA) and cytolytic toxins (such as aerolysin from Aeromonas hydrophila and maitotoxin) [31,37–39]. The NALP3-dependent inflammasome is also activated by cellular components that are released into the extracellular milieu by distressed cells such as crystals found in gout, namely monosodium urate and calcium pyrophosphate dihydrate [40]. Oral infection with *S.* Typhimurium of caspase-1–deficient mice, but not ASC^−/−^, NALP3^−/−^, or IPAF^−/−^ animals, leads to increased susceptibility to infection [41]. Similar observations have been reported with L. monocytogenes [42,43]. Furthermore, caspase-1– and ASC-deficient (but not IPAF- and NOD2-deficient) mice experienced increased susceptibility to Francisella tularensis, the agent of tularemia [44].

By using microinjection or liposome delivery or bacterial mutants, the caspase-1 activators, such as IpaB (from S. flexneri), SipB (from *S.* Typhimurium) and flagellin (from *S.* Typhimurium and Legionella pneumophila), have been recently proposed to signal through NAIP5 and IPAF [45–50]. Interestingly, NAIP5 is required to limit the maturation of phagosomes following in vitro infection by L. pneumophila, the agent of Legionnaires disease, [51] and restrict its intracellular replication independently of caspase-1 activation [49,52]. IPAF has similar functions that depend, however, on caspase-1 [52,53]. Notably, IPAF and caspase-1, but not ASC, are required to control pyroptosis and autophagy induced by Shigella independently of flagellin [54]. Whereas NAIP5 confers resistance to Legionnaires disease [55–57], the physiological role of IPAF remains elusive with respect to L. pneumophila. Unlike the inflammasome, the sensing of flagellin through TLR5 does not trigger caspase-1 activation, suggesting that IPAF and NAIP5 represent a fail-safe immune mechanism to respond to flagellated pathogens [48,57,58]. Lastly, interferon-β and tumor necrosis factor-α have been implicated in restricting the growth of L. pneumophila in macrophages, but independently of IPAF and NAIP5 [49,59]. Taken together, deficiency in NAIP5, ASC, and/or caspase-1 in mice are, instead, associated with increased susceptibility to invasive microbes and with resistance to the lethal effect of endotoxins [50,60]. Therefore, the NLR signaling pathway and the inflammasome represent “watchdog” machineries against both intra- and extracellular pathogens, including toxicogenic microbes.

## Optimal Immune Response Toward Pathogens Requires NLR

Given that several sensors are likely solicited when host cell faces a microorganism, the engagement of specific combinations of NLR and other PRM affect the host response by driving either tolerance, priming, or synergy. Notably, by using cell-based assays, it was found that chemically synthesized NOD1 and NOD2 agonists synergize TLR-dependent cytokines expression in monocytes [61–66]. Similar findings have been reported in dendritic cells (DCs) for the secretion of IL-12 [67,68], which is a major cytokine produced by DCs to promote T(H)1 polarization of T cells [69]. DCs are the central “professional” immune cells for the initiation of the adaptive immunity by activating the T lymphocyte differentiation into T(H)1 or T(H)2 cells. It is worth noting that the RIP2 signaling pathway is required for efficient elicitation of antigen-specific T and B cell immunity and for instructing subsequently the onset of T(H)1 and T(H)2, as well as T(H)17 immune pathways by regulating IL-23, IL-12, interferon-γ and IL-17 [13,18,70]. Conversely, NOD1- and NOD2-deficient bone marrow–derived macrophages and macrophages bearing loss-of-function of NOD2 failed to synergize the expression of inflammatory cytokines following concomitant stimulation by NOD1/2 and TLR agonists [13,14,63]. The role of the inflammasome in the development of autoimmune diseases and T(H)17 response to pathogens and “danger” signals remains to be further documented, because the IL-1 receptor is required to drive the development of experimental autoimmune encephalomyelitis and the production of IL-17 [71,72].

Triggering of a specific PRM induces a transient phenomenon of tolerance and/or cross-tolerance toward a second stimulation by the same agonist [73]. This unresponsiveness window may be essential to protect the host from sustained innate response. If a microorganism colonizes a TLR-responsive niche, one can expect that the TLR signaling might be exhausted. This TLR refractory state is nevertheless specific for this niche and for a certain period of time. In this context, subsequent signaling is likely to occur for unrepressed NLR signaling pathways [74,75]. Alternatively, sequential engagement of distinct TLR stimulates mainly the synergistic production of pro-inflammatory mediators [73]. Therefore, systematic dissection of the synergy and tolerance induced by TLR and/or NLR is now warranted for rationale therapeutic intervention.

The heterotypic interactions between NLR have also been proposed to regulate their function [76,77], as well as interplay with additional proteins like Erbin for the NOD2 signaling pathway [78]. Effective NALP1- and NALP3-dependent inflammasome activation requires both the synthesis of pro-IL-1β and several incoming signals. Activation of TLR signaling pathways has often been used as a first signal that drives the expression of the IL-1β–encoding gene [47–49,58,79–81]. Activation of caspase-1 and release of mature IL-1β might then result from the cytosolic detection by the inflammasome of a second signal (which are primarily microbial compounds and self-danger signals). Alternatively, the intracellular concentration of potassium and ATP/dATP, which might be modulated by toxins and pathogens, control inflammasome activation [80,82–84]. In this context, the transmembrane receptor Pannexin-1 and the P2X(7) receptor participate in the activation of the inflammasome by transferring bacterial components from outside to the cytosol [80]. It is worth noting the increased viability of “permissive” macrophages to replication of L. pneumophila, which is in part explained by a specific targeting of pro-death members of the Bcl2 protein family [85]. Similarly to what occurs in the nematode Caenorhabditis elegans, a complex interplay between cell survival and caspase-1 activation has been recently unraveled, as the mammalian anti-apoptotic proteins Bcl-2 and Bcl-Xl repress specifically the NALP1-mediated caspase-1 activation [35]. Implications for the pathophysiology of human inflammatory diseases and the pathogenesis of pathogens that activate caspase-1, like L. pneumophila, remain to be investigated for the development of rational prophylactic and anti-infectious therapies.

## NLR in Antimicrobial Immunity

Antimicrobial peptides, such as defensins, play an active role in fortifying the lining of the gut toward pathogens and commensals by preserving epithelium integrity and stem cell viability and by participating in the recruitment of immunocytes. Defensins are cationic antimicrobial peptides rich in cysteine residues that exert their activity by damaging the bacterial cell wall [86]. Mammalian defensins can be divided into two main subsets, the α and β defensins. In humans, α defensins 1–4 are produced by neutrophils and are stored in granules, whereas α defensins 5 and 6 (referred as cryptdins in mice) are mostly secreted by Paneth cells in the lumen of the small intestine. The human β defensins are more widely expressed and are synthesized by most of the epithelia. Proteases (e.g., trypsin and metalloproteinase-7) play an essential role in the maturation process of defensins [87,88]. Alternatively, the expression of certain antimicrobial peptide-encoding genes is down-regulated during shigellosis in humans and salmonellosis in mice [89,90]. In this context, we might speculate that specific combinations of PRM coordinate a three-steps immune response by regulating expression, degranulation, and maturation of antimicrobial peptides.

Recent evidences shed also lights on a nonredundant role of NLR and TLR in the differential expression and maturation of specific sets of antimicrobial peptides [91]. By using cell lines, NOD1 and NOD2 agonists have been shown to up-regulate the expression of the human β defensin-2 (hBD2)–encoding gene [91]. Likewise, inhibition of the NF-κB pathway totally blocks NOD1- and NOD2-dependent induction of hBD2 expression, whereas inhibition of p38 and JNK signaling pathways only partially diminish hBD2 expression in a NOD1-dependent manner [22,92]. Consistently, MDP-induced hBD2 expression was down-regulated after knocking down NOD2 or by transfecting HEK293 cells with the Crohn disease–associated 3020insC frameshift mutation [92]. In vivo, TLR5-deficient mice exhibit impaired expression of mouse β defensin-3 (the homolog of the hBD-2) by intestinal epithelial cells [93], whereas NOD1- and NOD2-deficient mice showed a deficiency in mouse β defensin-4 by gastric epithelial cells and in certain cryptdins by Paneth cells, respectively [13,22]. Combining experiments using mice and human models might shed light on the potential effect of antimicrobial peptides in response to specific infectious agents and in the control of physiological inflammation.

## Microbial Alteration of NLR Sensing and Signaling

Pathogens have also evolved strategies to circumvent their intracellular sensing through NLR such as NOD1 by preventing processing and optimal sensing of their PGN. Indeed, a *L. monocytogenes–*derived deacetylase, namely PgdA, is required to bypass the early innate immune response of NOD1 by specifically adding N-acetylglucosamine residues to the DAP-type PGN. *pgdA* mutants triggered sensitivity to the bacteriolytic activity of lysozyme, leading to increased NOD1-dependent interferon-β immune response and have impaired virulence in vivo [94]. Similarly, the PGN hydrolase AmiA is involved in the microbial pathogenesis of H. pylori by limiting the sensing by NOD1 [95]. The implications of post-translational changes of additional MAMP, including MDP, in microbial pathogenesis remain to be systematically investigated.

Commensal and symbiotic microorganisms may actively interfere with NLR-mediated response by down-regulating pro-inflammatory signaling and/or by modifying cell differentiation/proliferation. Bacteroides thetaiotamicron specifically stimulates the nucleus-cytosol shuttling of NF-κB in a peroxisome proliferator–activated receptor-γ–dependent mechanism [96]. The bacterial molecules driving this down-regulation might consequently affect NOD signaling. In addition to commensal, pathogenic bacteria are also able to interfere with NOD, as recently showed in *Yersinia* species that block stimulation of NF-κB– and MAPK-dependent gene expression through acetylation of MAPK [97]. In other respects, S. flexneri produces a phosphatase that is specific for nuclear MAPK, thus preventing histone H3 modification and NF-κB–dependent transcription of pro-inflammatory genes [98]. Lastly, modification of ubiquitination is also a candidate mechanism for invasive microbes to subvert sensing through NLR and is now the subject of investigation [99]. This conceptual view of regulation of NLR signaling now warrants further investigation to better understand the double-edged challenge of the mucosa toward commensals and pathogens in health and disease.

## Impaired TLR and NLR Function Are Sufficient to Drive Human Immunopathologies

Microbial sensing deficiency in fruit fly and mice confers an increased susceptibility to several infectious agents and leads to the development of autoimmune disease. In humans, defective NF-κB– and TLR-dependent immunity have been associated with restricted inherited infectious diseases, in that hypomorphic or null germline mutations of TLR5, IRAK4, NEMO, and UNC-93B are sufficient for the development of Legionnaires disease [100], recurrent pneumococcal disease [101], mycobacterial disease [102], and herpes simplex virus encephalitis [103], respectively. Similarly, common infection might result primarily from mutations in a major susceptibility gene, as impaired TLR2/Mal signaling is conferring protection against invasive pneumococcal disease, bacteremia, malaria, and tuberculosis in the United Kingdom, Vietnam, and several African countries through an increased frequency of the Ser allele at the Mal S180L variant [104]. Hence, these observations have challenged dogmas in the genetics of human infectious diseases, and they might suggest that both rare and common infectious diseases may result from defect in a major biological pathway.

Like infectious diseases, complex inflammatory traits result from the inheritance of major susceptibility alleles and the exposure to several environmental factors. No specific infectious agents have been identified so far, but note that gain-of-function mutations in NALP3 [105] and NOD2 [106] cause Mendelian inflammatory diseases, such as autoinflammatory disorders (Muckle-Wells syndrome, familial cold autoinflammatory syndrome, and chronic infantile neurologic cutaneous and articular syndrome) and Blau syndrome, respectively. Furthermore, predisposition to common inflammatory disorders has been inextricably linked to major susceptibility genes, as loss-of-function mutations in TLR5 and NOD2 are protecting and predisposing, respectively, to the development of systemic lupus erythematosus [107] and inflammatory bowel diseases [108,109]. Similarly, NEMO deficiency in enterocytes leads to the spontaneous development of colitis in mice by compromising tissue repair, epithelium integrity and promoting bacterial translocation [110]. A missense mutation (L155H) and noncoding polymorphism of the NALP1-encoding gene are predisposing to the development of vitiligo-associated multiple autoimmune disease [111], implicating innate immunity in the pathogenesis of autoimmunity. However, the constitutive and bacterial-induced level of caspase-1 activation remains elusive in cells bearing the NALP1 L155H mutation. Lastly, mutations in the gene encoding for NALP7 cause familial and recurrent hydatidiform moles [112–114], which are tumors that forms in the uterus as a mass of cysts resembling a bunch of grapes. Unlike NALP1-3, IPAF, and NAIP5, NALP7 is a negative regulator of IL-1β signaling [115] that promotes tumorigenesis [116]. Further work should now determine the following: (a) how the NALP7 signaling pathway might be activated, (b) whether NALP7 might interfere with additional PRM, and (c) whether NALP7 might regulate innate and adaptive immunity.

## Concluding Remarks

Whereas most studies have focused on the characterization of individual PRM, the physiological situation is more complex, because host cells have to integrate multiple incoming signals from damaged cells and pathogenic and symbiotic microbes into vital immunological information. In this context, organ-specific onset of innate and adaptive immunity is regulated differentially by multiple cross-talks between a limited set of functional PRM signaling pathways. 

## Abbreviations

ASC: apoptosis-associated speck-like protein containing a caspase recruitment domain
CARD: caspase-recruitment domain
DC: dendritic cell
ICE: IL-1–converting enzyme
IPAF: ICE protease-activating factor
LRR: leucine-rich repeat
MAMP: microbial-associated molecular patterns
MAPK: mitogen-activated protein kinase
MDP: muramyl dipeptide
MyD88: myeloid differentiation primary response gene (88)
NF-κB: nuclear factor–κ B
NLR: Nod-like receptors
NOD: nucleotide-binding oligomerization domain protein
PGN: peptidoglycan
PRM: pattern-recognition molecules
PYD: pyrin domain
TLR: Toll-like receptors
